# Supplementary material for: A comparison of the genes and genesets identified by GWAS and EWAS of fifteen complex traits
Source: Nat Commun. 2022 Dec 19;13:7816. doi: 10.1038/s41467-022-35037-3 (PMC9763500; doi:10.1038/s41467-022-35037-3)
Supplement: Supplementary file 1 — Supplementary Information [file 41467_2022_35037_MOESM1_ESM.pdf]

Supplementary Information
A comparison of the genes and genesets identified by EWAS and GWAS of fifteen complex traits

Thomas Battram[1,2]*, Tom R. Gaunt[1,2], Caroline L. Relton[1,2], Nicholas J. Timpson[1,2], Gibran Hemani[1,2]
[1] MRC Integrative Epidemiology Unit, University of Bristol, UK
[2] Population Health Sciences, Bristol Medical School, University of Bristol, UK

This document contains supplementary figures 1-7 and a supplementary note on how power to detect associations between DNA methylation and complex traits varies when the nature of association is causal, confounded, or reverse causal.

*Corresponding author: thomas.battram@bristol.ac.uk

Figures

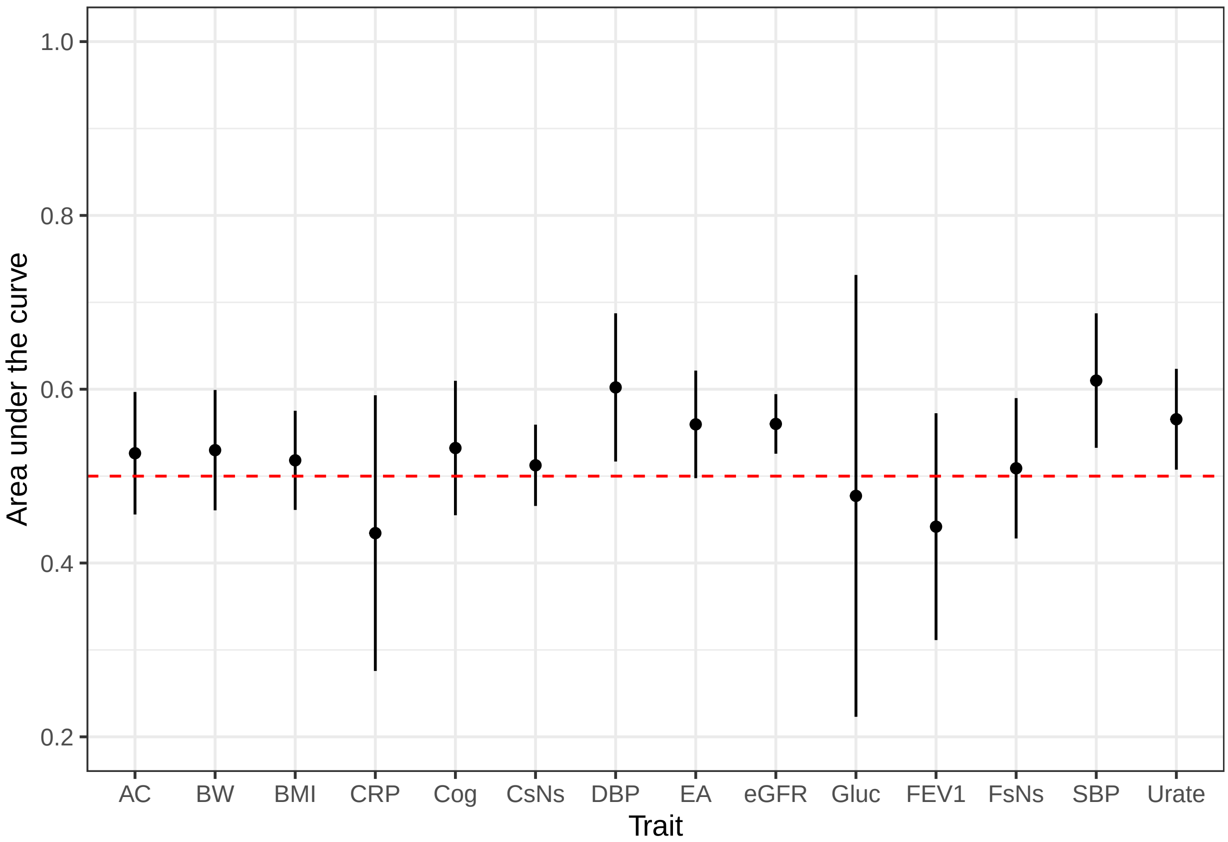

Figure 1: **Can genetic variant associations predict the presence of DNA methylation associations in the same region?.** The genome was divided into 500kb regions. Those where no probes on the HM450 array measured DNAm were excluded from the analysis. This left 5591 regions. For each 500kb region in the genome, the largest SNP-trait effect size was extracted. ROC curves were produced to determine whether these could predict whether a differentially methylated position related to the same trait was present in the same 500kb region. The point estimate for the area under these curves (AUC), with their confidence intervals, are plotted for each trait. The red dashed line is at AUC = 0.5, which represents a prediction no better than chance. List of traits with the sample sizes of the GWAS and EWAS in the form of trait (GWAS N; EWAS N): AC = alcohol consumption per day (335,394; 9643), BW = birthweight (143,677; 8825), BMI = body mass index (681,275; 10,238), CKD = chronic kidney disease, Cog = cognitive ability (digit test) (257,841; 4794), CRP = c-reactive protein (204,402; 8863), CsNs = current smokers vs never smokers (632,802; 9389), DBP = diastolic blood pressure (757,601; 17,010), EA = educational attainment (766,345; 10,767), eGFR (961,734; 33,605) Gluc = fasting glucose (58,074; 4808), FEV1 = forced expiratory volume in one second (421,986; 5370), FsNs = former smokers vs never smokers (424,960; 13,474), SBP = systolic blood pressure (757,601; 17,010), Urate (278,592; 17,996). Note: insulin is missing from this plot as there was no overlap between identified DNAm sites and genetic variants in any 500kb region and so an AUC could not be calculated.

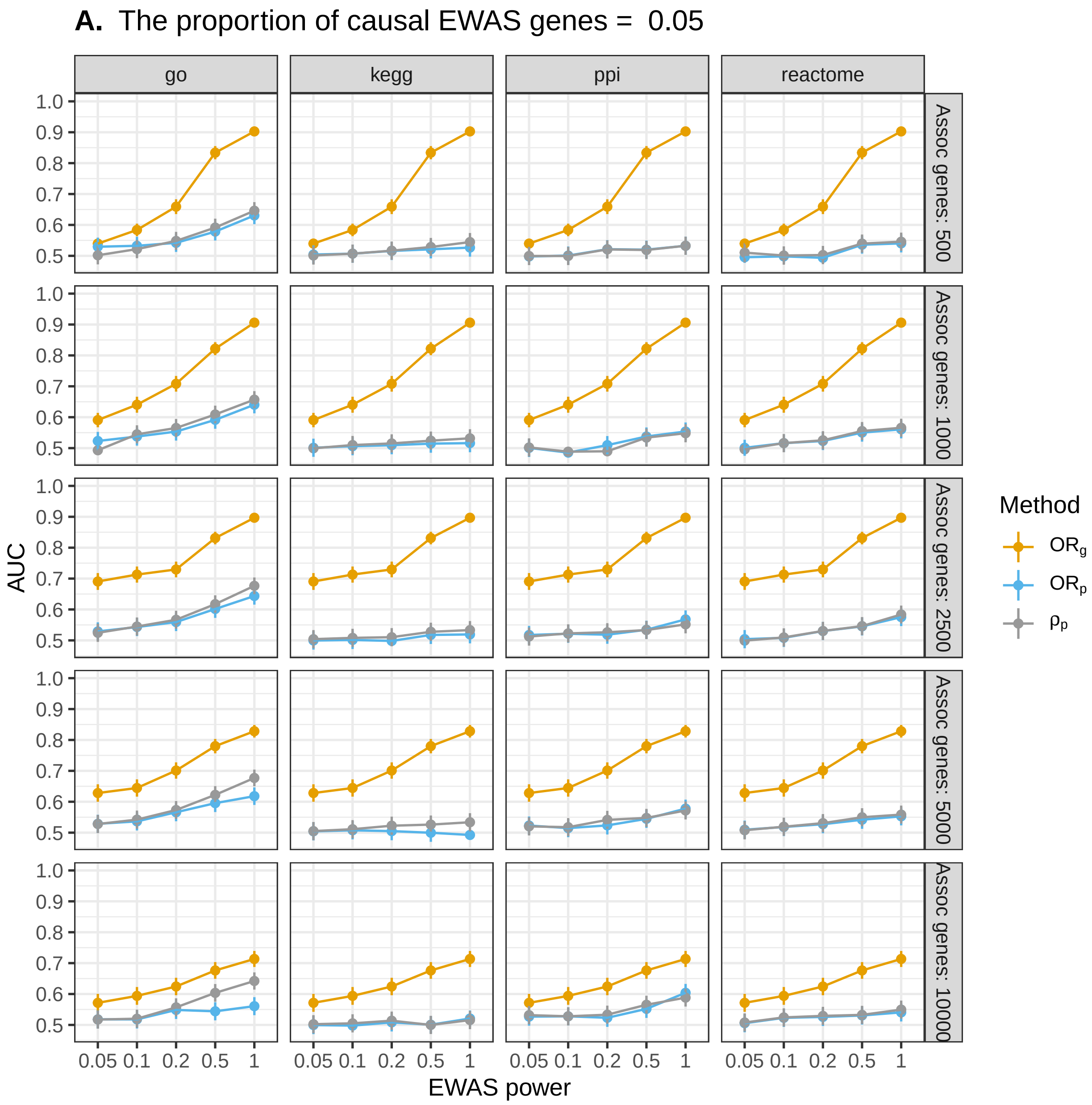

A. The proportion of causal EWAS genes = 0.05
go
kegg
ppi
reactome
Assoc genes: 500
Assoc genes: 1000
Assoc genes: 2500
Assoc genes: 5000
Assoc genes: 10000
AUC
1.0
0.9
0.8
0.7
0.6
0.5
0.05 0.1 0.2 0.5 1
EWAS power
Method
OR_g
OR_p
ρ_p

9

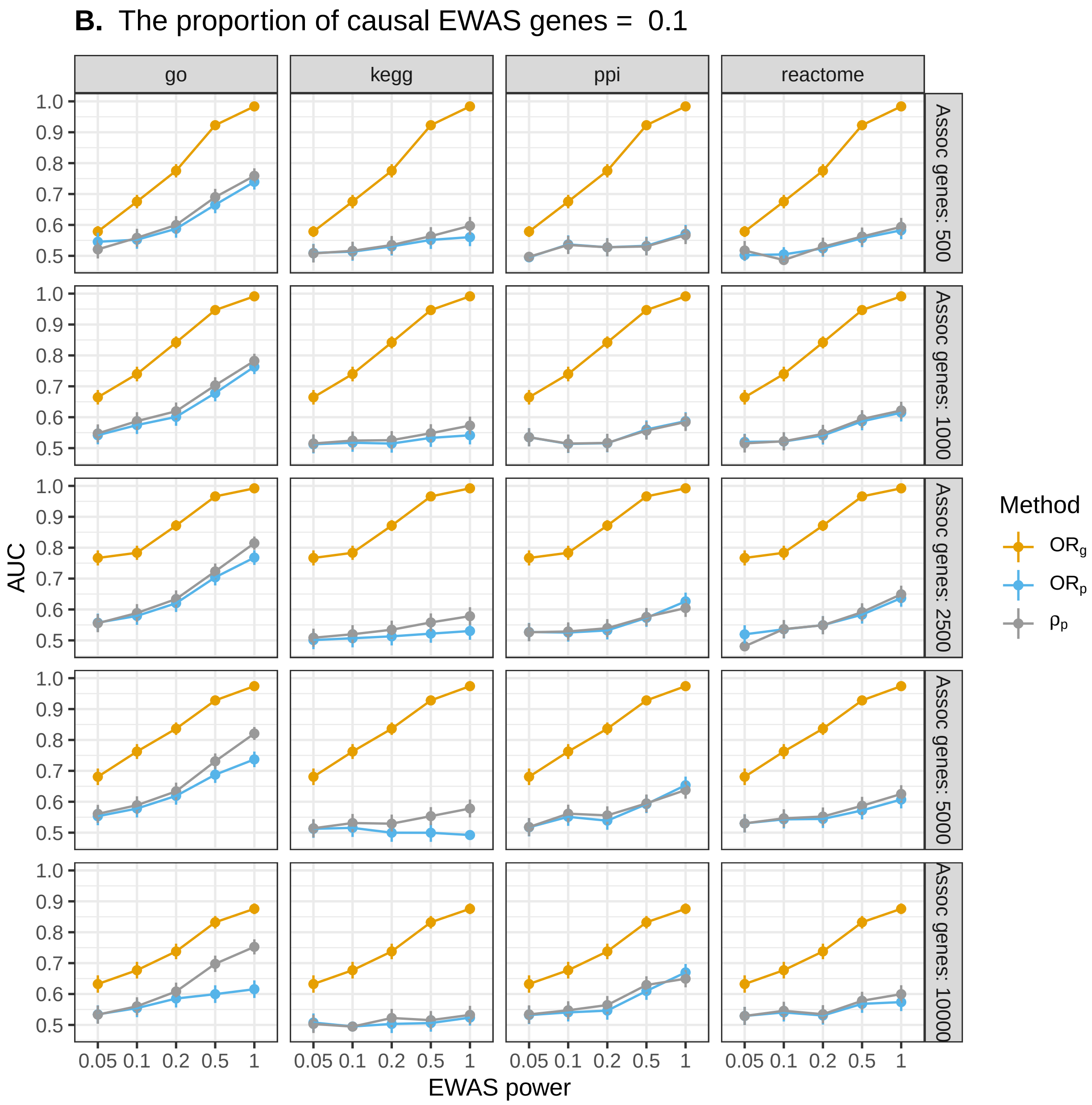
B. The proportion of causal EWAS genes = 0.1
go
kegg
ppi
reactome
Assoc genes: 500
Assoc genes: 1000
Assoc genes: 2500
Assoc genes: 5000
Assoc genes: 10000
AUC
1.0
0.9
0.8
0.7
0.6
0.5
0.05 0.1 0.2 0.5 1
EWAS power
Method
ORg
ORp
ρp

10

C. The proportion of causal EWAS genes = 0.2

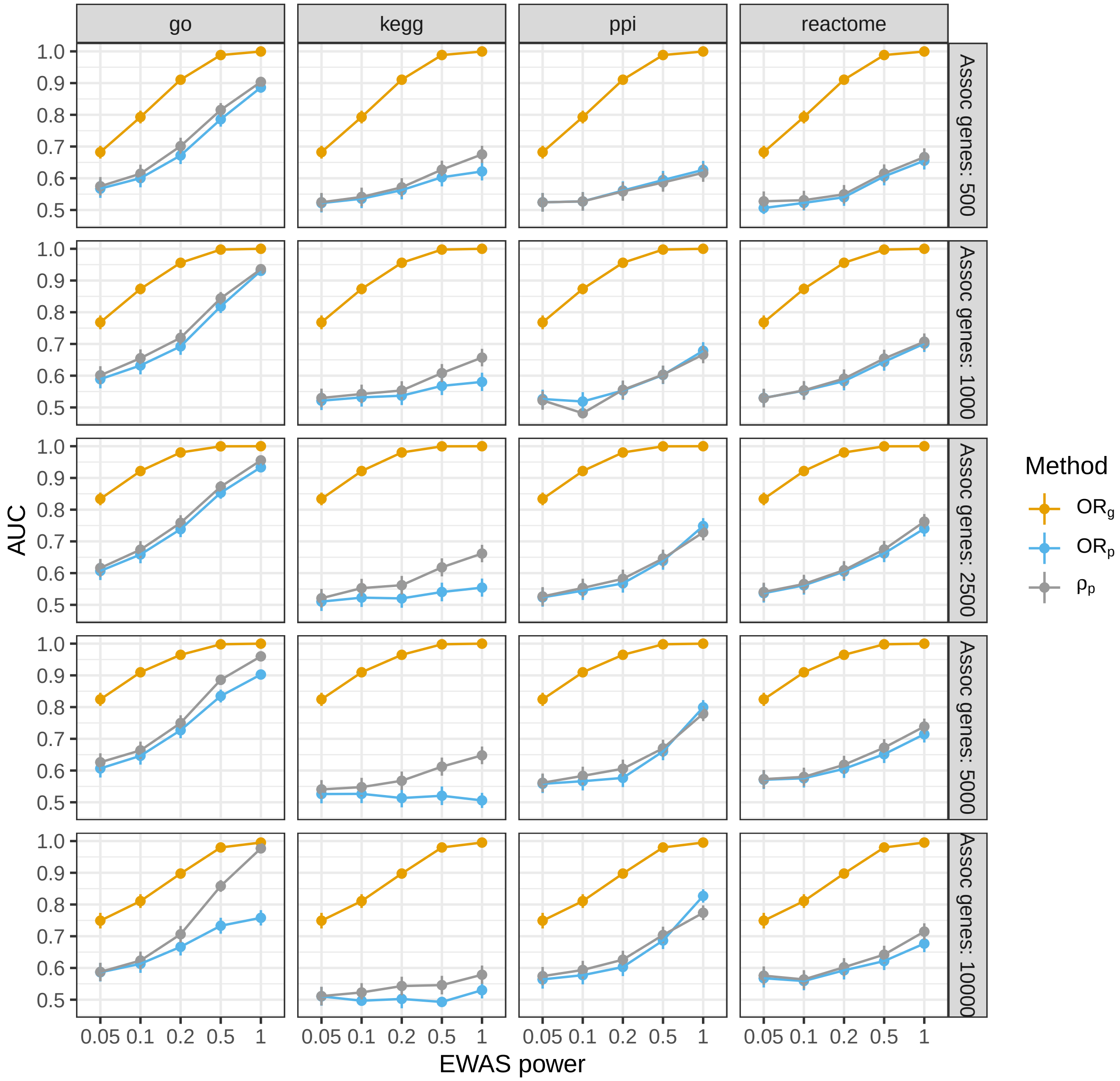

11

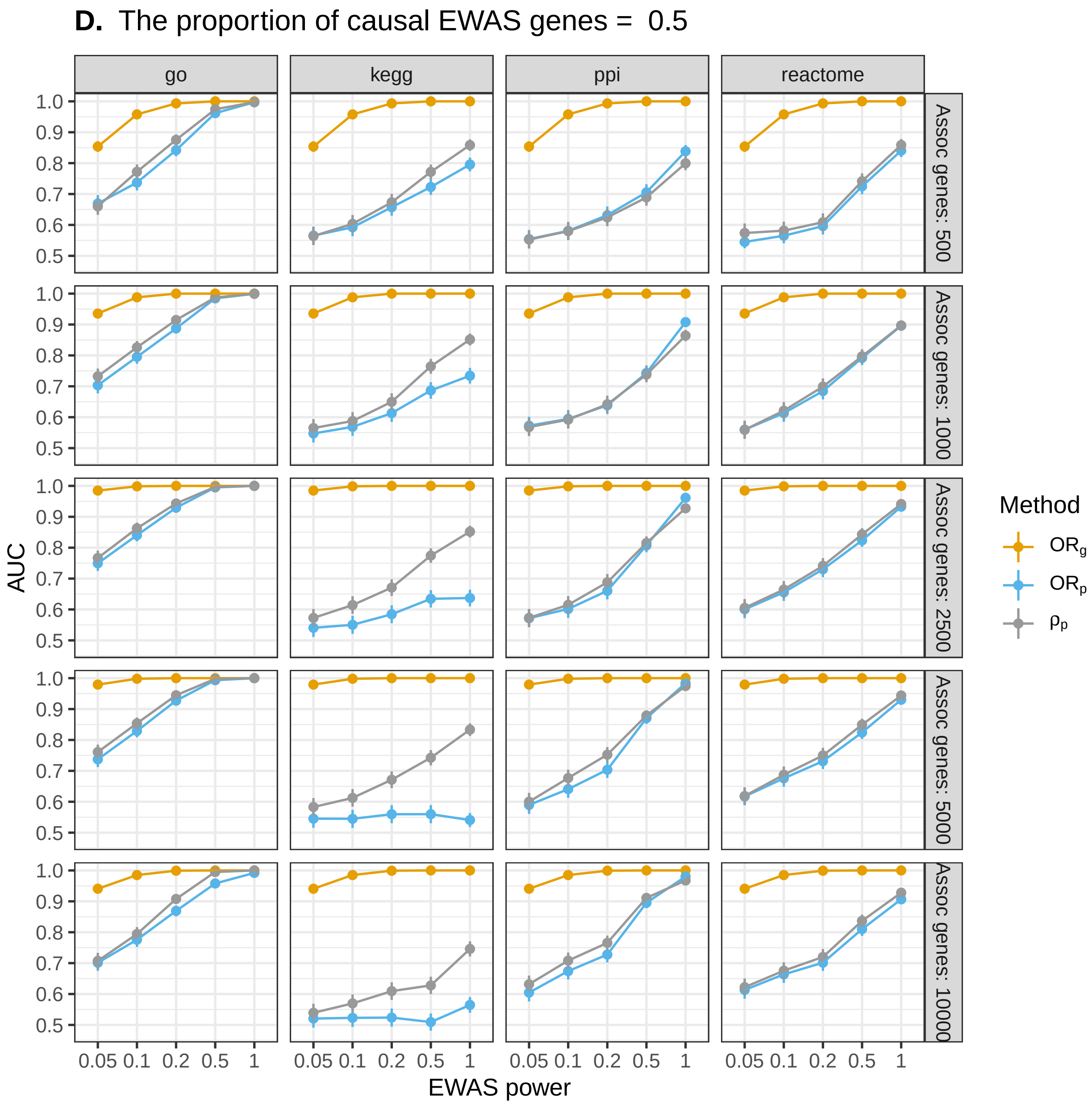
D. The proportion of causal EWAS genes = 0.5
go
kegg
ppi
reactome
Assoc genes: 500
Assoc genes: 1000
Assoc genes: 2500
Assoc genes: 5000
Assoc genes: 10000
AUC
EWAS power
Method
OR_g
OR_p
ρ_p

12

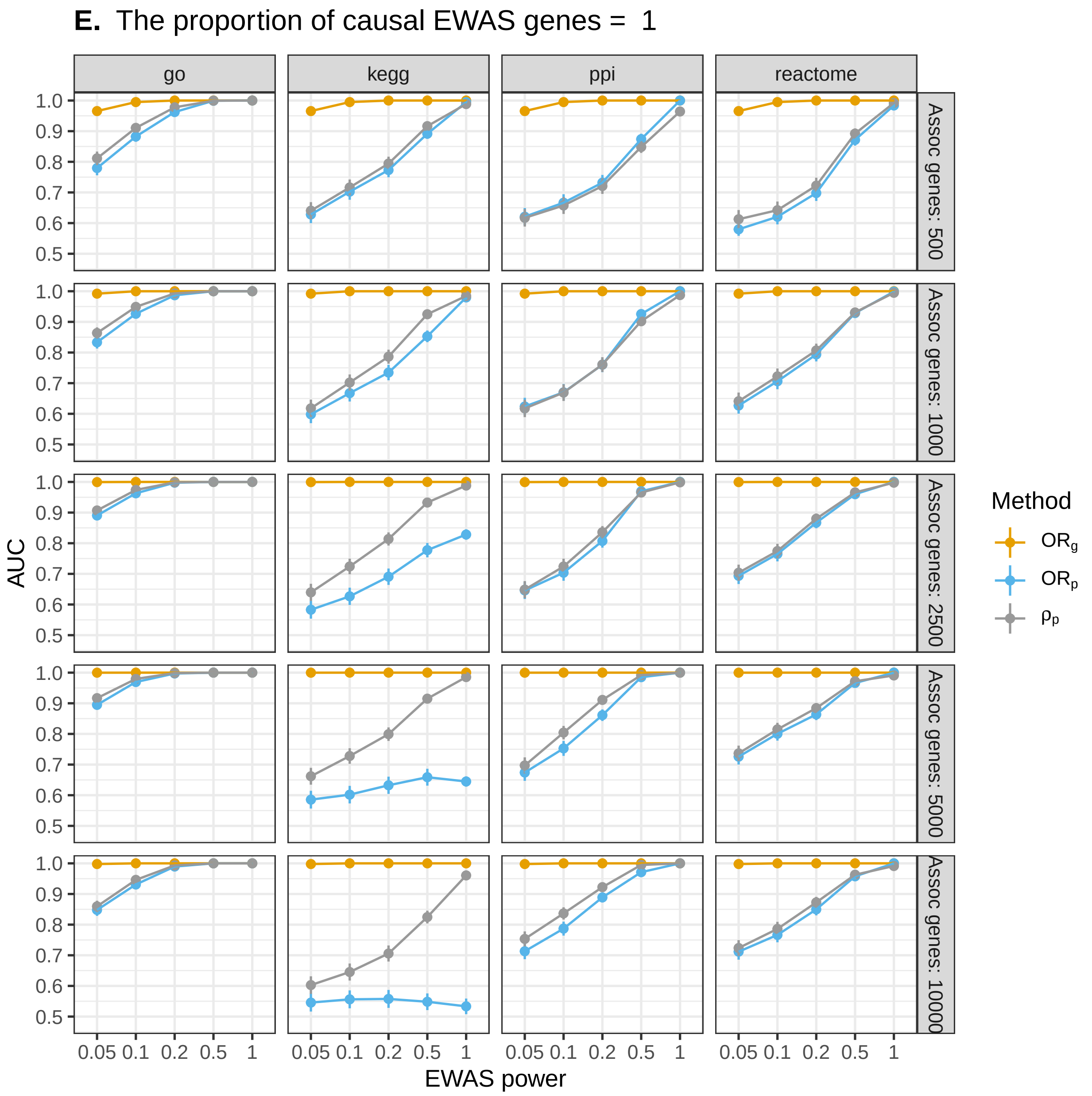

Figure 2: **Power to detect overlap between genes and genesets identified by corresponding EWAS and GWAS.** Simulations were set up as illustrated in **Box 1**. EWAS power is equivalent to the proportion of associated genes (Assoc genes) EWAS is detecting. In the scenario where Assoc genes = 500, EWAS power = 1, and the proportion of causal EWAS genes = 0.05, the EWAS is detecting 500 genes, 25 of which are causal. Panels A-E show results across an increasing proportion of causal EWAS genes (A = 0.05, B = 0.1, C = 0.2, D = 0.5, E = 1). The area under receiver operator curves (AUC) was used to estimate the ability to distinguish between results generated when GWAS and EWAS were sampling, in part, from the same set of causal genes and results generated when EWAS was sampling random genes from the genome. Error bars represent the 95% confidence intervals of the AUC estimates. The header of each set indicates the proportion of genes identified by the simulated EWAS that were set to be causal. $\mathrm{OR_g}$ = assessing overlap of genes, $\mathrm{OR_p}$ = assessing overlap of genesets, ρ_p = assessing correlation between geneset enrichment scores. GO = gene ontology, PPI = protein-protein interaction database from EpiGraphDB.

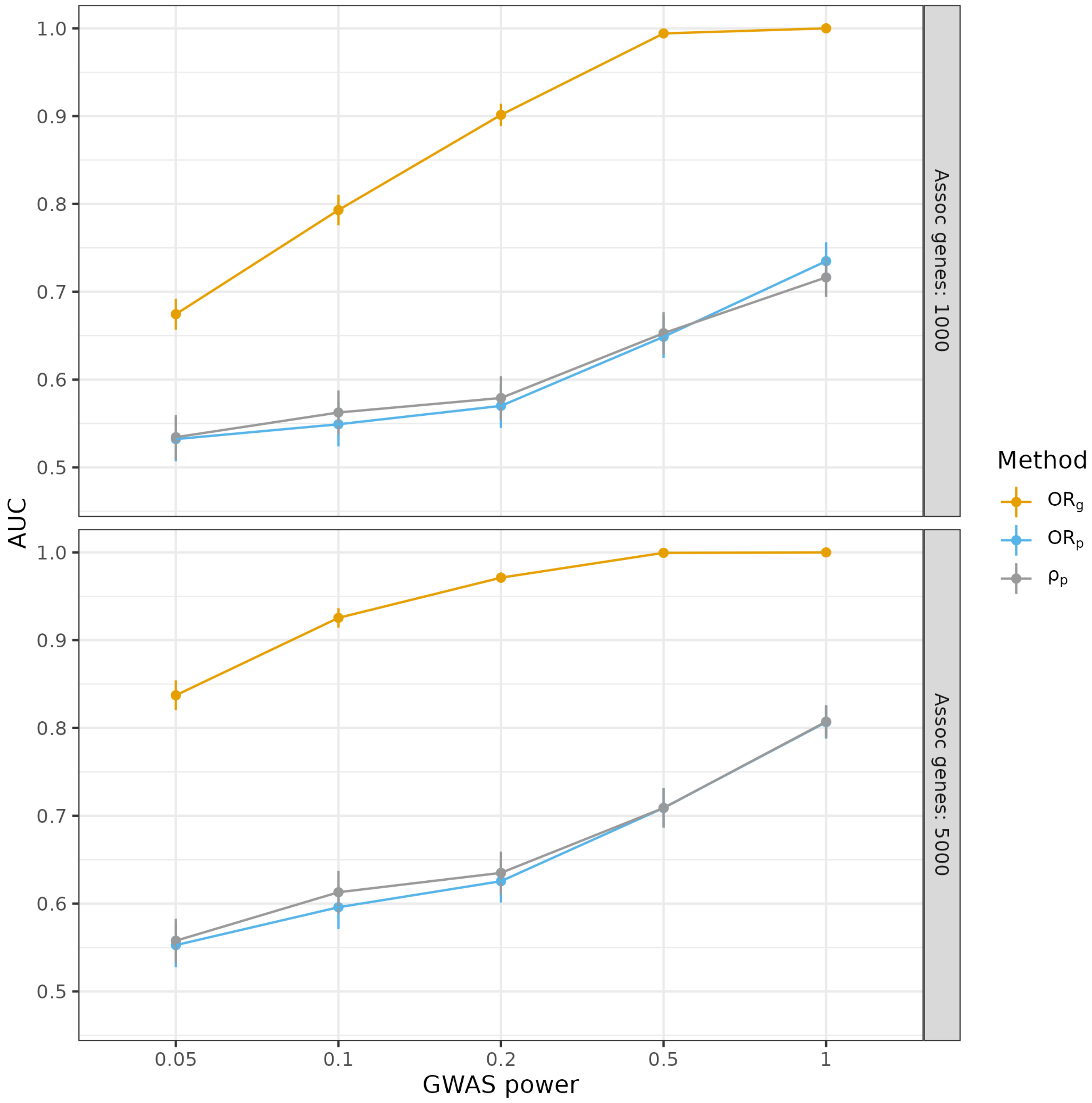

Figure 3: **Power to detect overlap between genes and genesets identified by corresponding EWAS and GWAS.** Simulations were set up described in **Box 1**. The ability to distinguish between results generated when EWAS and GWAS were sampling, in part, from the same set of causal genes and results generated when EWAS was sampling random genes from the genome. This figure shows how power to detect overlap changes with GWAS power changes, between methods and when different numbers of genes are used. EWAS power was kept at 0.1 and the proportion of genes identified by EWAS that were set to be causal was 0.1. Error bars represent the 95% confidence intervals of the AUC estimates. $\mathrm{OR_g}$ = assessing overlap of genes, $\mathrm{OR_p}$ = assessing overlap of genesets, ρ_p = assessing correlation between geneset enrichment scores.

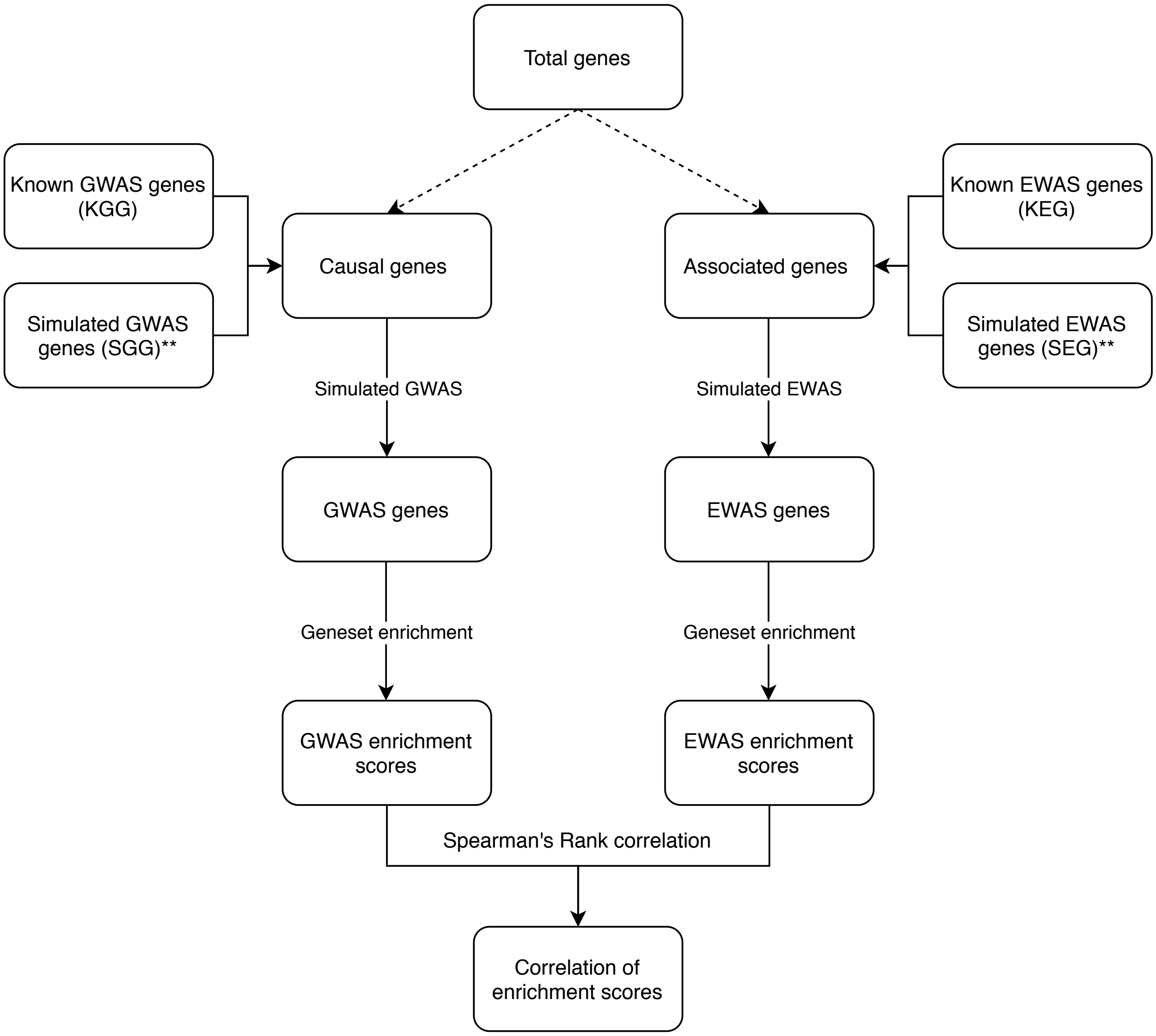

Figure 4: **Flowchart demonstrating how the second set of simulations were set up for each trait.** Phenotypic variation will be caused by changes in gene/protein polymers (causal genes) and can be associated with changes in gene/protein polymers via other routes such as confounding or reverse causation (associated genes). In these simulations the causal genes were a mix of genes identified by GWAS of that trait, known GWAS genes (KGG), and a randomly selected set of genes, simulated GWAS genes (SGG). The associated genes were a mix of genes identified by EWAS of that trait, known EWAS genes (KEG), and a randomly selected set of genes, simulated EWAS genes (SEG). The level of overlap in the causal and associated genes was modified by changing the overlap in the SGG and SEG. The number of causal and associated genes was kept the same for each simulation, but this number varied between simulations. The minimum number of causal genes and the minimum number of associated genes was equal to the sum of KGG and KEG. The “simulated GWAS” step in the simulation simply equates to randomly sampling from the causal genes. The number of genes sampled was equal to the number of KGG. The “simulated EWAS” step was identical except the number of KEG from the associated genes. Geneset enrichment was performed as described in the **Methods**. The simulations were repeated 1000 times for each set of parameters.

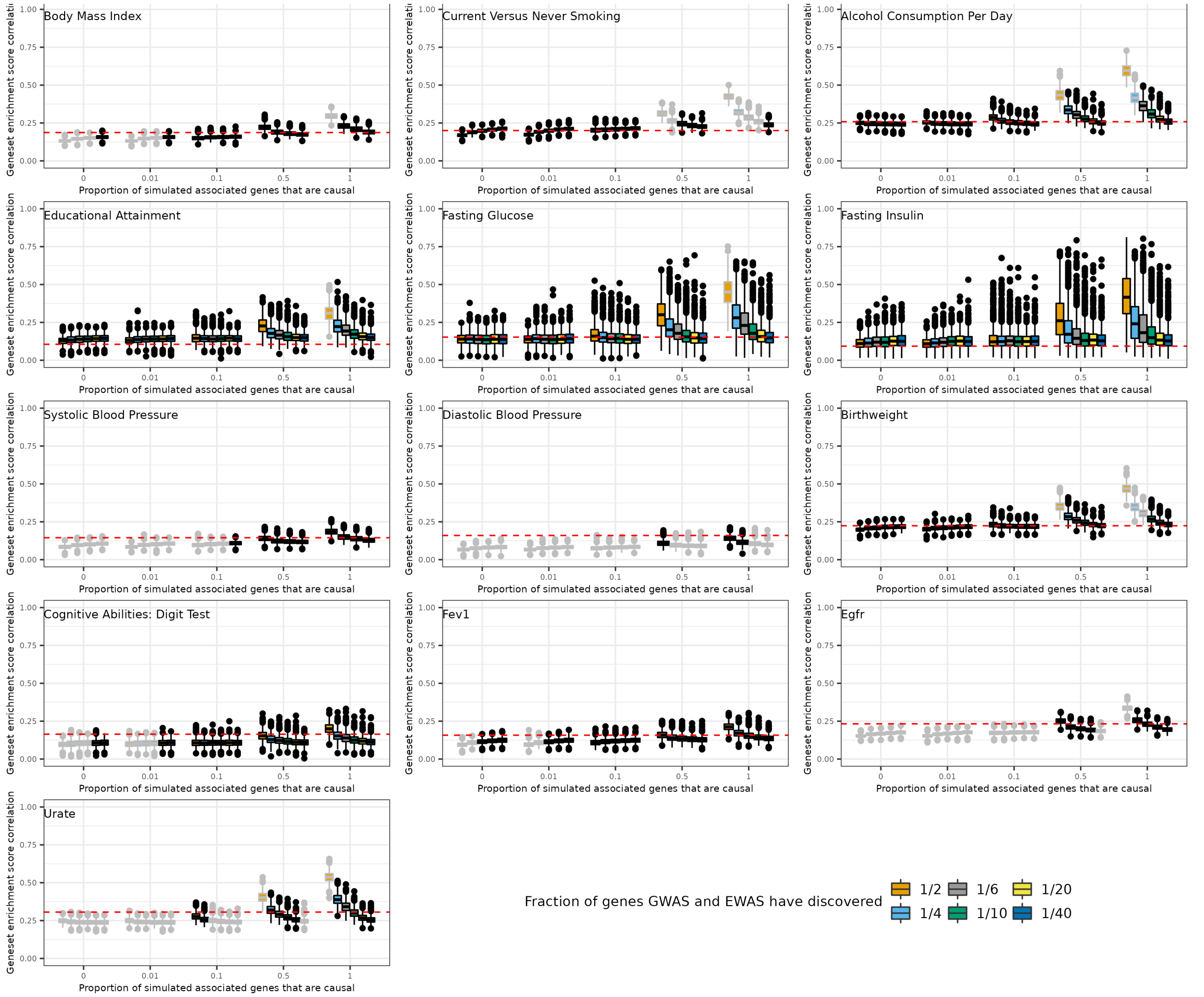

Figure 5: **Simulations to understand the likely number of genes still to identify in EWAS and GWAS of 13 traits under different trait architectures**. Simulations were set up as illustrated in **Supplementary figure 4**. Correlation of geneset enrichment scores from empirical data (**Table 3**), is shown as a red dashed line. Box plots show the range of enrichment score correlations from 1000 simulations using the parameters indicated. The number of causal and associated genes, as well as the number of associated genes that were causal were varied. Already discovered EWAS genes were added to the pool of associated genes and already discovered GWAS genes were added to the pool of causal genes. The proportion of simulated associated genes that were causal is shown on the X-axis. The number of causal genes and associated genes were equal in each simulation. Scenarios which lie close to the empirical result (red dashed line) are more likely to reflect the true underlying number of genes related to a trait and the true overlap between the causal and associated genes. Where there is evidence that geneset enrichment scores from a simulation scenario are close to the empirical enrichment score (FDR < 0.05, z-test for difference), the box outline is black, otherwise it is grey. The centre of the box plots are the median, the bounds of the box represent the interquartile range (IQR), the upper whisker represents either the minimum of (1.5 multiplied by the IQR) + the 75% percentile and the maximum value, the lower whisker represents the maximum of 25% percentile - (1.5 multiplied by the IQR) and the maximum value. Values that fall outside the whiskers are marked as points.

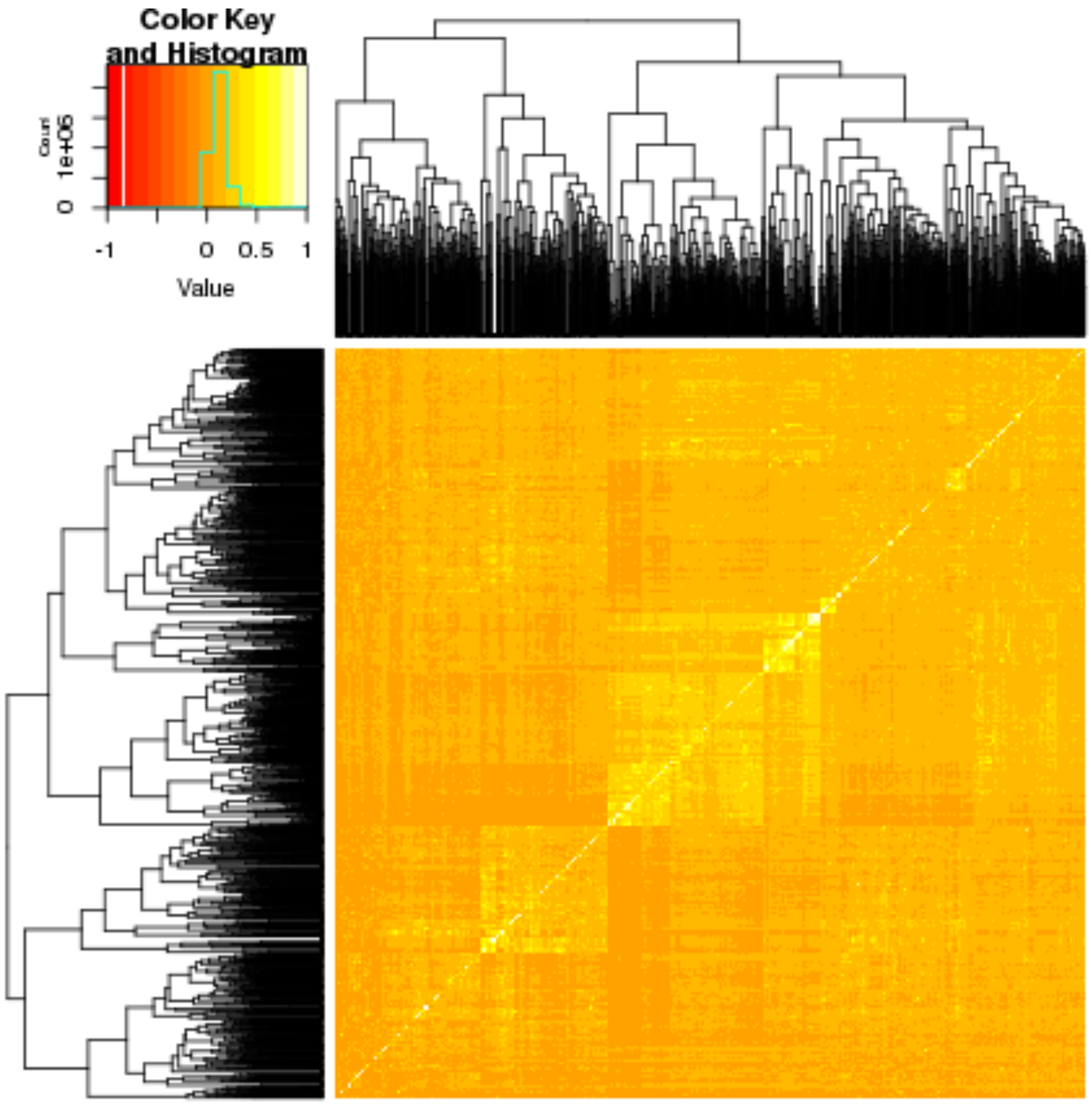

Figure 6: **Correlation across geneset enrichment scores for 1886 GWAS and 15 EWAS.**

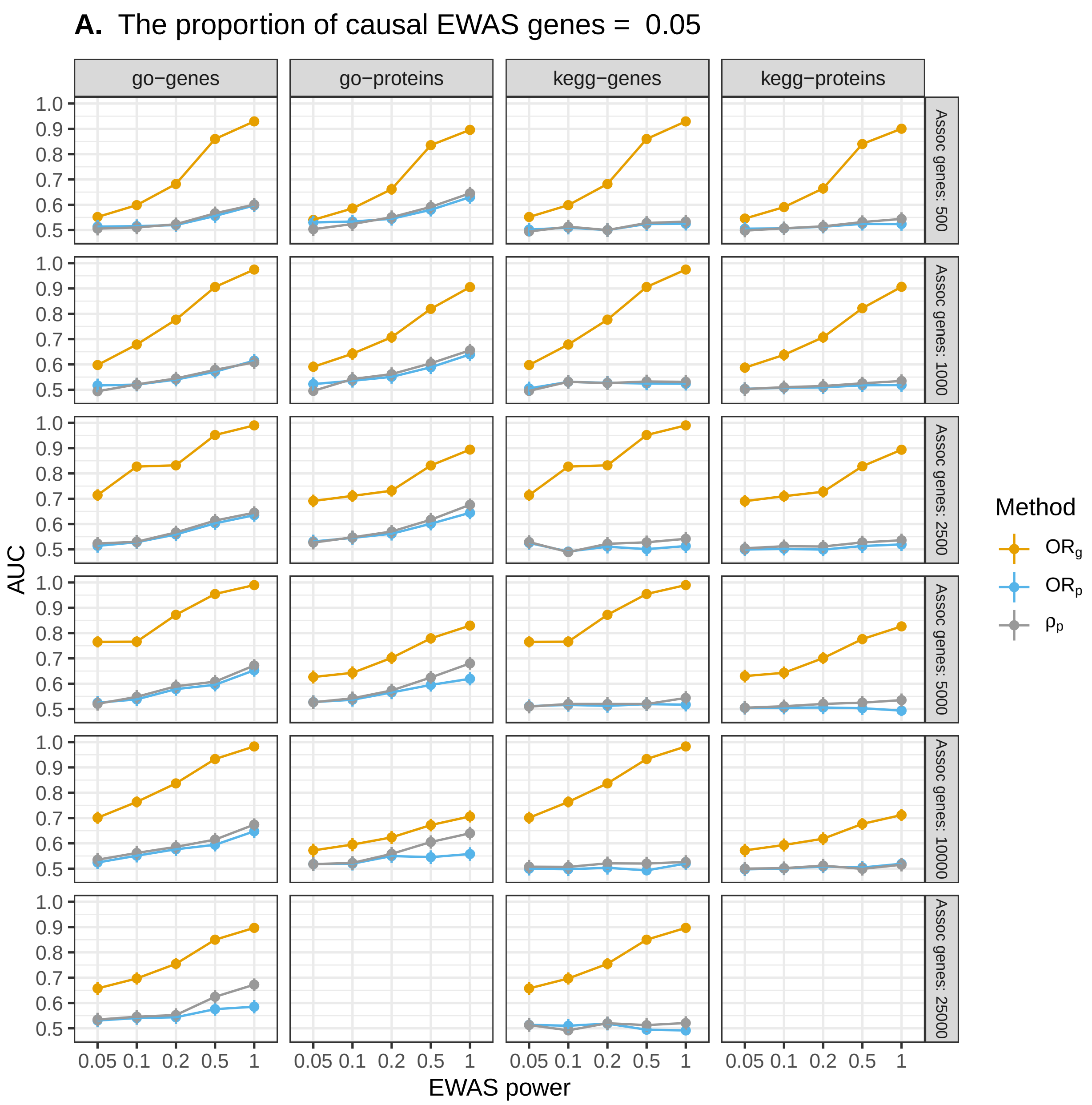
A. The proportion of causal EWAS genes = 0.05
go−genes
go−proteins
kegg−genes
kegg−proteins
Assoc genes: 500
Assoc genes: 1000
Assoc genes: 2500
Assoc genes: 5000
Assoc genes: 10000
Assoc genes: 25000
AUC
EWAS power
Method
OR_g
OR_p
ρ_p

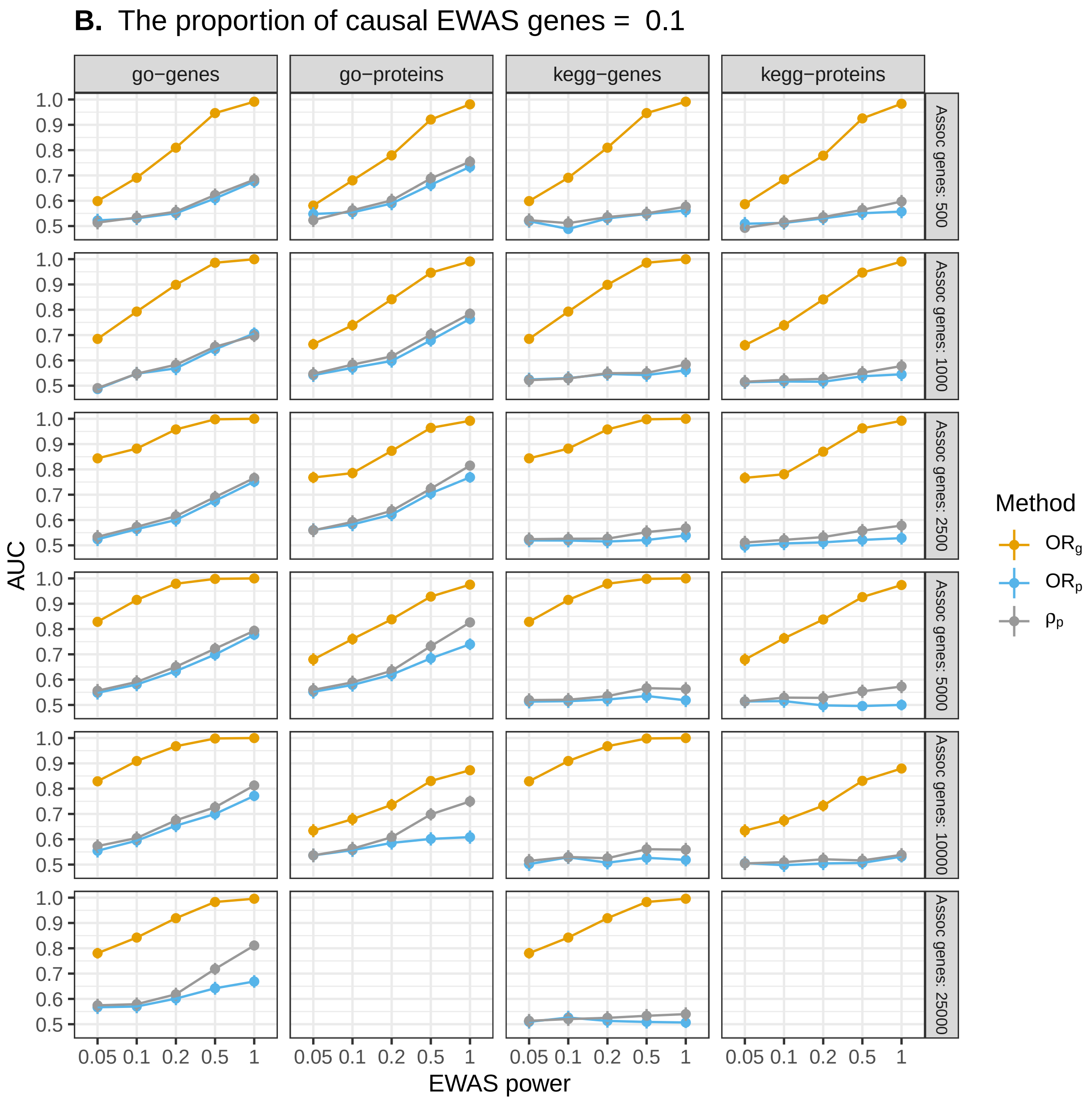
B. The proportion of causal EWAS genes = 0.1
go−genes
go−proteins
kegg−genes
kegg−proteins
Assoc genes: 500
Assoc genes: 1000
Assoc genes: 2500
Assoc genes: 5000
Assoc genes: 10000
Assoc genes: 25000
AUC
1.0
0.9
0.8
0.7
0.6
0.5
0.05 0.1 0.2 0.5 1
EWAS power
Method
OR_g
OR_p
ρ_p

C. The proportion of causal EWAS genes = 0.2

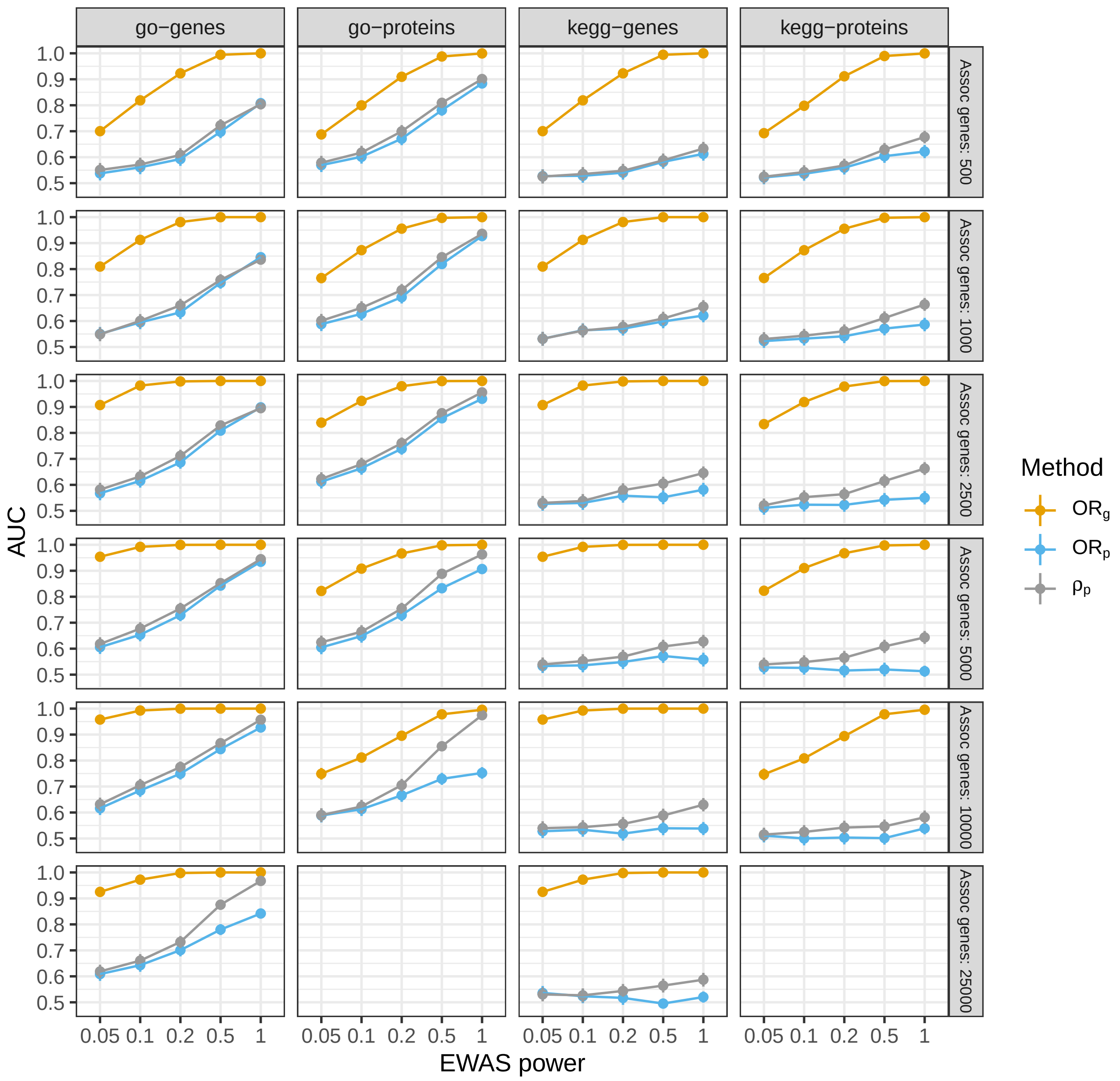

15

D. The proportion of causal EWAS genes = 0.5

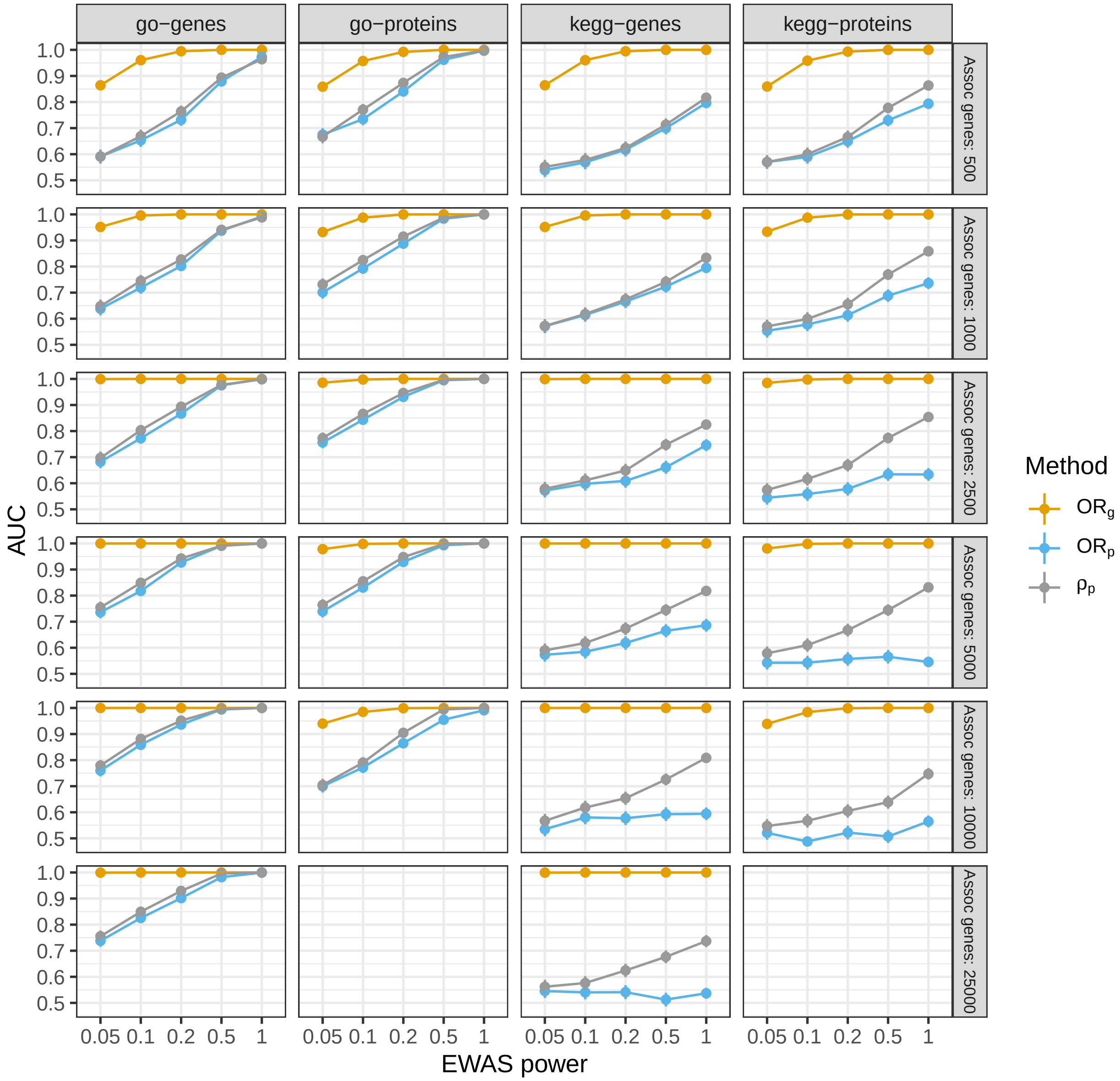

16

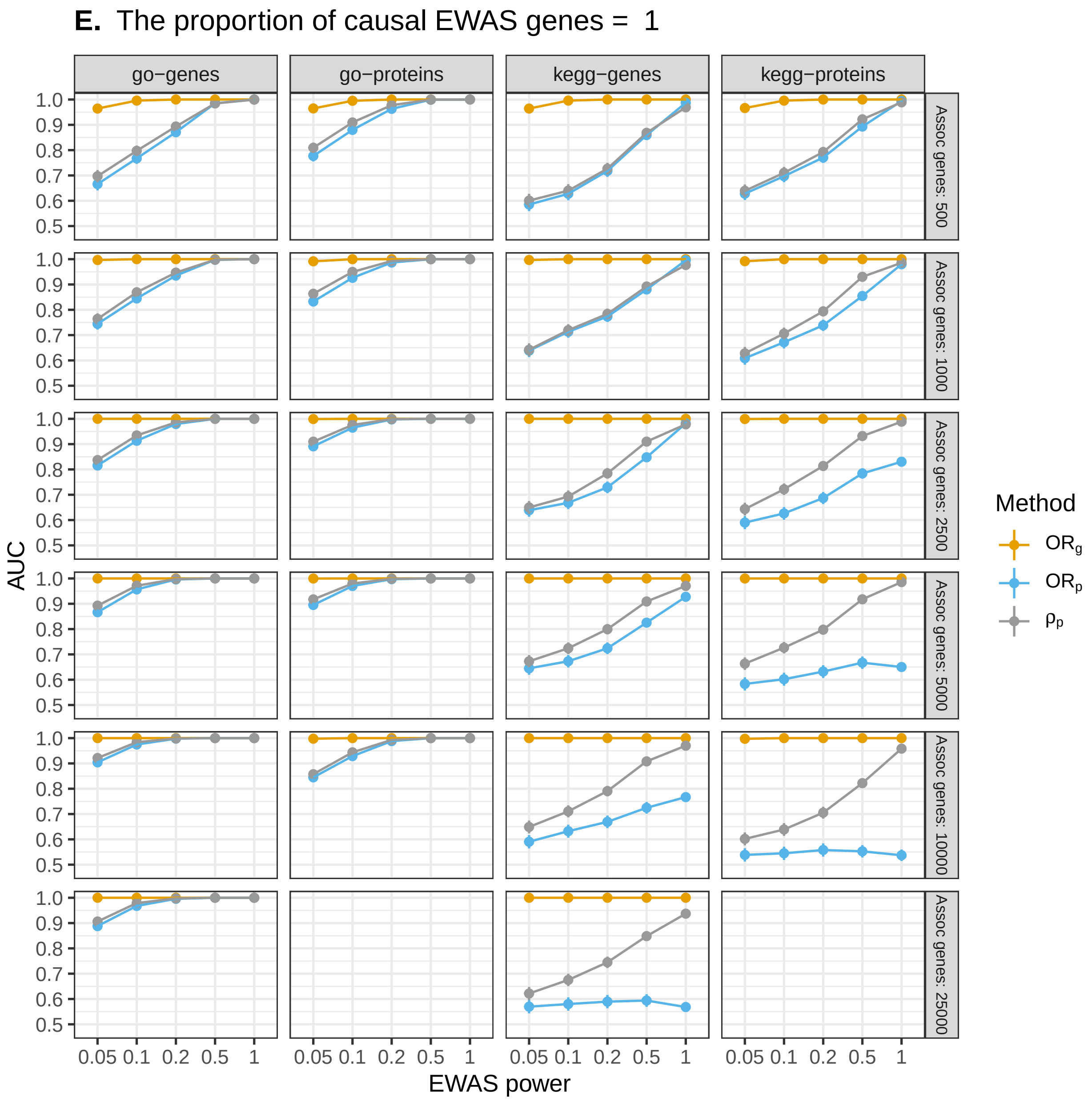

Figure 7: **Power to detect overlap between genes and genesets identified by corresponding EWAS and GWAS when mapping signal to all genes and protein coding genes.** Simulations were set up as illustrated in **Box 1**. EWAS power is equivalent to the proportion of associated genes (Assoc genes) EWAS is detecting. In the scenario where Assoc genes = 500, EWAS power = 1, and the proportion of causal EWAS genes = 0.05, the EWAS is detecting 500 genes, 25 of which are causal. Panels A-E show results across an increasing proportion of causal EWAS genes (A = 0.05, B = 0.1, C = 0.2, D = 0.5, E = 1). The area under receiver operator curves (AUC) was used to estimate the ability to distinguish between results generated when GWAS and EWAS were sampling, in part, from the same set of causal genes and results generated when EWAS was sampling random genes from the genome. Error bars represent the 95% confidence intervals of the AUC estimates. The header of each set indicates the proportion of genes identified by the simulated EWAS that were set to be causal. $\mathrm{OR_g}$ = assessing overlap of genes, $\mathrm{OR_p}$ = assessing overlap of genesets, ρ_p = assessing correlation between geneset enrichment scores. GO = gene ontology, PPI = protein-protein interaction database from EpiGraphDB. A suffix of '-genes' denotes using all Ensembl gene IDs for the analysis and the suffix of '-proteins' denotes using only protein coding genes.

Supplementary Note: Epigenome-wide association study architecture and power

Introduction

Epigenome-wide association study (EWAS) associations in which DNA methylation levels correlate with complex trait variation can arise due to (1) forward causality (whereby DNA methylation changes cause trait variation), (2) reverse causality (whereby trait variation causes change in DNA methylation levels) or (3) confounding. Each of these models is subject to different constraints under a multi-factorial model of complex traits. Suppose that our complex trait y has P_y factors and a methylation site m_j is influenced by P_{m_j} factors. Though methylation sites are known to be multi-factorial themselves, in general we would consider that $P_y \gg P_{m_j}$.

If you have P factors that influence a value y then they have the following constraint

$$R_T^2 = \sum_{j=1}^{P} R_{m_j y}^2 \leq 1$$

where R_T^2 = the total variance of a trait and $R_{m_j y}^2$ = the variance explained by methylation site j.

In principle the variance not captured by $R_{m_j y}^2$ is basically stochastic noise. Therefore the larger the value of P, the smaller the value that $R_{m_j y}^2$ can take, and the lower the power to detect any factor for y. Suppose that $Var(y) = 1$ and $Var(m_j) = 1$ then $\beta_{m_j y} \sim N(0, R_T^2/P)$.

Below we discuss the implication for discovery of CpG sites in EWAS when associations arise due to forward causality, reverse causality, and confounding.

Models

Forward causal

If all CpG-trait associations are due to CpGs being causal then they are amongst the P_y causal factors for y then the effects are constrained to be drawn from $\beta_{m_j y} \sim N(0, R^2_{T_y}/P_y)$. So $R^2_{m_j y}(1) = \beta^2_{m_j y}$.

Reverse causal

If all CpG-trait associations are reverse causal, then each CpG is independently influenced by y with an effect of $\beta_{ym_j} \sim N(0, R^2_{T_{m_j}}/P_{m_j})$. So $R^2_{m_j y}(2) = \beta^2_{ym_j}$

Confounded

If all CpG-trait associations are confounded then each CpG is independently influenced by some confounder u that also influences y. So the effect of u on m_j is subject to the constraint in (2) and the effect of u on y is subject to the constraint in (1).

$$\begin{aligned}
R^2_{m_j y}(3) &= \frac{Cov(m_j, y)^2}{Var(m_j)Var(y)} \\
&= Cov(\beta_{um_j} u, \beta_{uy} u)^2 \\
&= Var(u)^2 \beta^2_{um_j} \beta^2_{uy} \\
&= \beta^2_{um_j} \beta^2_{uy}
\end{aligned}$$

Assume the variance of u is 1, overall the expected association will be $R^2_{m_j y}(3) = \beta^2_{uy}\beta^2_{um_j}$.

Expected number of associations in EWAS

Statistical power of EWAS is mostly related to variance in the trait explained by the CpG ($R^2_{m_j y}$) and the sample size (N). For P_y DNA methylation sites that relate to y the number expected to be associated is the sum of the power across all sites.

Using the models above as a guide, below are simulations demonstrating how power to detect forward causal, reverse causal, and confounded associations in EWAS differ under various scenarios.

Simulations

Generate a function that will estimate the expected number of associations for each of the three models

```
suppressMessages(suppressPackageStartupMessages({
library(pwr)
library(dplyr)
library(ggplot2)
}))
#' Calculate power to detect an EWAS association under models of forward causality, revers
#'
#' @param Pm causal factors influencing DNAm
#' @param Py causal factors influencing the trait (Y)
#' @param R2m variance explained in DNAm by the trait
#' @param R2y variance explained in the trait by DNAm
#' @param N sample size
#' @param thresh P value threshold for a "significant" association
#'
#' @return tibble of input parameters and number of expected associations
calc_power <- function(Pm, Py, R2m, R2y, N, thresh)
{
  # Model 1 (forward causal)
  b1 <- rnorm(Py, mean=0, sd=sqrt(R2y/Py))
  pow1 <- pwr.r.test(N, b1, thresh)$power
  nsig1 <- sum(pow1)

  # Model 2 (reverse causal)
  b2 <- rnorm(Py, mean=0, sd=sqrt(R2m/Pm))
  pow2 <- pwr.r.test(N, b2, thresh)$power
  nsig2 <- sum(pow2)

  # Model 3 (confounded)
  b3 <- b1 * b2
  pow3 <- pwr.r.test(n=N, r=b3, sig.level=thresh)$power
  nsig3 <- sum(pow3)

  return(tibble(Pm=Pm, Py=Py, R2m=R2m, R2y=R2y, N=N, thresh=thresh,
                model=c("Forward causal", "Reverse causal", "Confounded"),
                nsig=c(nsig1, nsig2, nsig3)
  ))
}
```

Set the parameters across which the simulations will run

```
param <- expand.grid(
  Pm = c(5, 50, 500),
  Py = seq(500, 10000, by=500),
  R2m = c(0.02, 0.2, 0.8),
  R2y = c(0.3, 0.5, 0.7),
  N = c(1000, 10000, 100000),
  thresh=5e-7
)
res <- lapply(1:nrow(param), function(i) do.call(calc_power, param[i,])) %>% bind_rows()
```

Visualise the expected yield of associations from each model

```r
subset(res, R2m == 0.2 & R2y == 0.5) %>%
ggplot(., aes(x=Py, y=nsig)) +
  geom_line(aes(colour=as.factor(model))) +
  facet_grid(N ~ Pm, labeller=label_both) +
  scale_y_log10() +
  geom_hline(yintercept=1, linetype="dotted") +
  scale_colour_brewer(type="qual") +
  labs(x="Number of causal factors for Y (Py)",
       y="Expected number of associations",
       colour="Model") +
  theme_bw() +
  theme(legend.position="bottom")
```

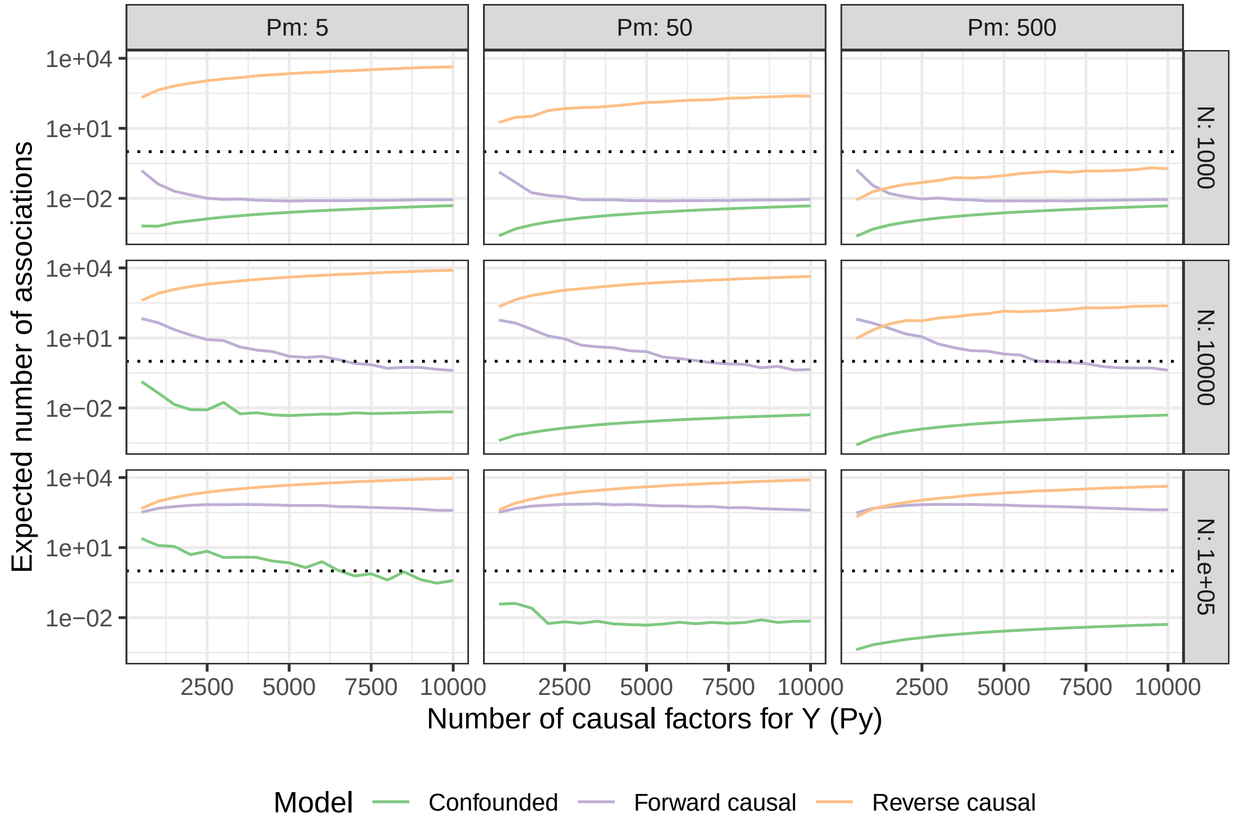

Figure 8: Each box represents the expected number of EWAS associations (y-axis, note the log scale) against how multi-factorial the trait is (x-axis) for the given parameters under each of the three causal models. Rows of boxes represent different sample sizes, and columns of boxes represent how multi-factorial the DNAm sites are. The black dotted line depicts the location on the y-axis for detecting a single causal variant, drawn for convenience.

Summary

In general, the power to detect EWAS associations whereby the complex trait of interest influences DNA methylation at a given site (reverse causal), greatly exceeds the power to detect DNA methylation changes that effect the trait (forward causal) when the trait is substantially more multi-factorial than each of the DNA methylation sites. EWAS typically have the least power to detect associations arising due to confounding, though this assumes that each confounder has a small effect on the trait (y).
