# Supplementary material for: A comparison of the genes and genesets identified by GWAS and EWAS of fifteen complex traits
Source: Nat Commun. 2022 Dec 19;13:7816. doi: 10.1038/s41467-022-35037-3 (PMC9763500; doi:10.1038/s41467-022-35037-3)
Supplement: Supplementary file 2 — Description of Additional Supplementary Files [file 41467_2022_35037_MOESM2_ESM.pdf]

Description of Additional Supplementary Files

File Name: Supplementary Data 1
Description: Traits, sample sizes (N), and PubMed IDs from 1886 GWAS extracted from the IEU OpenGWAS Project.
