# Supplementary material for: A comparison of the genes and genesets identified by GWAS and EWAS of fifteen complex traits
Source: Nat Commun. 2022 Dec 19;13:7816. doi: 10.1038/s41467-022-35037-3 (PMC9763500; doi:10.1038/s41467-022-35037-3)
Supplement: Supplementary file 4 — Reporting Summary [file 41467_2022_35037_MOESM4_ESM.pdf]

nature portfolio

Corresponding author(s): Thomas Battram

Last updated by author(s): Nov 10, 2022

Reporting Summary

Nature Portfolio wishes to improve the reproducibility of the work that we publish. This form provides structure for consistency and transparency in reporting. For further information on Nature Portfolio policies, see our Editorial Policies and the Editorial Policy Checklist.

Statistics

For all statistical analyses, confirm that the following items are present in the figure legend, table legend, main text, or Methods section.

n/a	Confirmed	
☐	☒	The exact sample size (*n*) for each experimental group/condition, given as a discrete number and unit of measurement
☒	☐	A statement on whether measurements were taken from distinct samples or whether the same sample was measured repeatedly
☐	☒	The statistical test(s) used AND whether they are one- or two-sided<br>*Only common tests should be described solely by name; describe more complex techniques in the Methods section.*
☒	☐	A description of all covariates tested
☐	☒	A description of any assumptions or corrections, such as tests of normality and adjustment for multiple comparisons
☐	☒	A full description of the statistical parameters including central tendency (e.g. means) or other basic estimates (e.g. regression coefficient) AND variation (e.g. standard deviation) or associated estimates of uncertainty (e.g. confidence intervals)
☐	☒	For null hypothesis testing, the test statistic (e.g. *F*, *t*, *r*) with confidence intervals, effect sizes, degrees of freedom and *P* value noted<br>*Give P values as exact values whenever suitable.*
☒	☐	For Bayesian analysis, information on the choice of priors and Markov chain Monte Carlo settings
☒	☐	For hierarchical and complex designs, identification of the appropriate level for tests and full reporting of outcomes
☐	☒	Estimates of effect sizes (e.g. Cohen's *d*, Pearson's *r*), indicating how they were calculated

Our web collection on statistics for biologists contains articles on many of the points above.

Software and code

Policy information about availability of computer code

Data collection	All the data used in this analysis is publicly available. Thirteen out of fifteen EWAS summary statistics can be downloaded from The EWAS Catalog (www.ewascatalog.org). The eGFR EWAS summary statistics was downloaded from https://ckdgen.imbi.uni-freiburg.de/files/Schlosser2021/eGFR.csv.zip and the urate summary statistics was downloaded from https://ckdgen.imbi.uni-freiburg.de/files/Tin2021/urate.csv.zip. Thirteen of fifteen GWAS summary statistics can be downloaded from the OpenGWAS Project (https://gwas.mrcieu.ac.uk/). The eGFR GWAS summary statistics were downloaded from https://ckdgen.imbi.uni-freiburg.de/files/Stanzick2021/metal_eGFR_meta_ea1.TBL.map.annot.gc.gz and the urate GWAS summary statistics were downloaded from https://ckdgen.imbi.uni-freiburg.de/files/Tin2019/urate_chr1_22_LQ_IQ06_mac10_EA_60_prec1_nstud30_summac400_rsid.txt.gz.
Data analysis	Code used to run the analyses is available here: https://zenodo.org/badge/latestdoi/521364771. The code is also on GitHub: https://github.com/thomasbattram/ewas-gwas-comparisons<br>Analyses were completed using R (version 3.6.2) and Python (version 3.7.4).

For manuscripts utilizing custom algorithms or software that are central to the research but not yet described in published literature, software must be made available to editors and reviewers. We strongly encourage code deposition in a community repository (e.g. GitHub). See the Nature Portfolio guidelines for submitting code & software for further information.

Data

Policy information about availability of data

All manuscripts must include a data availability statement. This statement should provide the following information, where applicable:

- Accession codes, unique identifiers, or web links for publicly available datasets
- A description of any restrictions on data availability
- For clinical datasets or third party data, please ensure that the statement adheres to our policy

All the data used in this analysis is publicly available. Thirteen EWAS summary statistics can be downloaded from The EWAS Catalog (http://ewascatalog.org/) under accession codes: 28002404_body_mass_index_discovery_and_replication, 27651444_smoking_current_vs_never_smoking, 27651444_smoking_former_vs_never_smoking, 27843151_alcohol_consumption_per_day_european_ancestry, 27955697_creactive_protein_discovery, 29086770_educational_attainment_basic_model, 31197173_Liu-J_fasting_glucose_base, 31197173_Liu-J_fasting_insulin_base, 29198723_Richard-MA_systolic_blood_pressure_meta-analysis, 29198723_Richard-MA_diastolic_blood_pressure_meta-analysis, 31015461_Kupers-L_birthweight_meta_analysis_all_ancestries, 29311653_Marioni-R_cognitive_abilities__digit_test_basic_adjusted_model, 31073081_Imboden-M_fev1_meta-analysis. The two EWAS not included here are for eGFR and urate. The eGFR EWAS summary statistics was downloaded from https://ckdgen.imbi.uni-freiburg.de/files/Schlosser2021/eGFR.csv.zip and the urate summary statistics was downloaded from https://ckdgen.imbi.uni-freiburg.de/files/Tin2021/urate.csv.zip. The GWAS summary statistics can be downloaded from the OpenGWAS Project (https://gwas.mrcieu.ac.uk/) under accession codes: ieu-b-40, ieu-b-4877, ukb-b-2134, ieu-b-73, ieu-b-35, ieu-a-1239, ebi-a-GCST005186, ebi-a-GCST005185, ieu-b-38, ieu-b-39, ieu-a-1083, ebi-a-GCST006572, ukb-b-19657. The eGFR GWAS summary statistics were downloaded from https://ckdgen.imbi.uni-freiburg.de/files/Stanzick2021/metal_eGFR_meta_ea1.TBL.map.annot.gc.gz and the urate GWAS summary statistics were downloaded from https://ckdgen.imbi.uni-freiburg.de/files/Tin2019/urate_chr1_22_LQ_IQ06_mac10_EA_60_prec1_nstud30_summac400_rsid.txt.gz.

The R package biomaRt (v2.50.3) was used to extract Ensembl gene ids along with chromosome positions of all genes. The package was also used to extract gene ontology (GO) terms and map these to the Ensembl gene ids. The R package limma (v3.50.3) was used to extract KEGG terms and these were mapped to Ensembl gene ids.

Protein-protein interaction data, which includes data from StringDB and IntAct, and terms from the Reactome database were extracted from EpiGraphDB (v1.0) (http://www.epigraphdb.org/).

Human research participants

Policy information about studies involving human research participants and Sex and Gender in Research.

Reporting on sex and gender	N/A
Population characteristics	N/A
Recruitment	N/A
Ethics oversight	N/A

Note that full information on the approval of the study protocol must also be provided in the manuscript.

Field-specific reporting

Please select the one below that is the best fit for your research. If you are not sure, read the appropriate sections before making your selection.

☒ Life sciences ☐ Behavioural & social sciences ☐ Ecological, evolutionary & environmental sciences

For a reference copy of the document with all sections, see nature.com/documents/nr-reporting-summary-flat.pdf

Life sciences study design

All studies must disclose on these points even when the disclosure is negative.

Sample size	We selected traits for which EWAS had been conducted with over 4500 samples, had more than 10 associations at P < 1x10-7 and for which corresponding well-powered GWAS (N > 50000) summary data were available. Sample sizes were chosen so we had sufficient associations to make comparisons between the study types.
Data exclusions	All GWAS were conducted in European populations. EWAS were conducted in European populations only, or were part of trans-ancestry meta-analyses, that contained European individuals and heterogeneity analyses were conducted within each study showing high correlation of associations between populations. Traits were also excluded from analyses if they had fewer than 10 EWAS associations at P < 1x10-7.
Replication	Findings not replicated as data not available.

Randomization	Not relevant to study as we used only summary statistics available to us.
Blinding	Not relevant to study as we used only summary statistics available to us.

Reporting for specific materials, systems and methods

We require information from authors about some types of materials, experimental systems and methods used in many studies. Here, indicate whether each material, system or method listed is relevant to your study. If you are not sure if a list item applies to your research, read the appropriate section before selecting a response.

Materials & experimental systems

n/a	Involved in the study
☒	☐ Antibodies
☒	☐ Eukaryotic cell lines
☒	☐ Palaeontology and archaeology
☒	☐ Animals and other organisms
☒	☐ Clinical data
☒	☐ Dual use research of concern

Methods

n/a	Involved in the study
☒	☐ ChIP-seq
☒	☐ Flow cytometry
☒	☐ MRI-based neuroimaging
